# Glutamine Metabolism and Prostate Cancer

**DOI:** 10.3390/cancers16162871

**Published:** 2024-08-18

**Authors:** Holger H. H. Erb, Nikita Polishchuk, Oleh Stasyk, Uğur Kahya, Matthias M. Weigel, Anna Dubrovska

**Affiliations:** 1 Department of Urology, Technische Universität Dresden, 01307 Dresden, Germany; holger.erb@uniklinikum-dresden.de; 2 Department of Cell Signaling, Institute of Cell Biology, National Academy of Sciences of Ukraine, 79000 Lviv, Ukraine; polishchuk@cellbiol.lviv.ua (N.P.); stasyk@cellbiol.lviv.ua (O.S.); 3 OncoRay-National Center for Radiation Research in Oncology, Faculty of Medicine and University Hospital Carl Gustav Carus, Technische Universität Dresden and Helmholtz-Zentrum Dresden-Rossendorf, 01309 Dresden, Germany; u.kahya@hzdr.de (U.K.); matthias.weigel@ukdd.de (M.M.W.); 4 Helmholtz-Zentrum Dresden-Rossendorf, Institute of Radiooncology-OncoRay, 01328 Dresden, Germany; 5 German Cancer Research Center (DKFZ), 69120 Heidelberg, Germany; 6 German Cancer Consortium (DKTK), Partner Site Dresden, 01309 Dresden, Germany; 7 National Center for Tumor Diseases (NCT), Partner Site Dresden, 01307 Dresden, Germany

**Keywords:** glutamine, prostate cancer, therapy resistance, radiation therapy, chemotherapy, androgen receptor

## Abstract

**Simple Summary:**

Glutamine (Gln) plays a critical role in the development and progression of prostate cancer (PCa). Non-malignant prostate cells are not addicted to Gln, but PCa cells become Gln-dependent due to the low levels of the Gln-producing enzyme glutamine synthetase. Gln metabolism in PCa is controlled by oncogenes such as MYC, AR, and mTOR, which contribute to therapy resistance and more aggressive forms of the disease. Studies have shown that depriving PCa cells of Gln can inhibit their growth and survival and make them more sensitive to radiotherapy. Targeting Gln metabolism is, therefore, a promising approach for PCa treatment. Clinical trials are currently testing the safety and efficacy of drugs that inhibit Gln metabolism in combination with other therapies for different types of cancer, including PCa. Understanding of how tumor cells metabolically interact with their environment may help improve these treatments and patient outcomes. In this review, we present the latest advances in Gln research in PCa and insights into the current clinical trials.

**Abstract:**

Glutamine (Gln) is a non-essential amino acid that is involved in the development and progression of several malignancies, including prostate cancer (PCa). While Gln is non-essential for non-malignant prostate epithelial cells, PCa cells become highly dependent on an exogenous source of Gln. The Gln metabolism in PCa is tightly controlled by well-described oncogenes such as MYC, AR, and mTOR. These oncogenes contribute to therapy resistance and progression to the aggressive castration-resistant PCa. Inhibition of Gln catabolism impedes PCa growth, survival, and tumor-initiating potential while sensitizing the cells to radiotherapy. Therefore, given its significant role in tumor growth, targeting Gln metabolism is a promising approach for developing new therapeutic strategies. Ongoing clinical trials evaluate the safety and efficacy of Gln catabolism inhibitors in combination with conventional and targeted therapies in patients with various solid tumors, including PCa. Further understanding of how PCa cells metabolically interact with their microenvironment will facilitate the clinical translation of Gln inhibitors and help improve therapeutic outcomes. This review focuses on the role of Gln in PCa progression and therapy resistance and provides insights into current clinical trials.

## 1. Glutamine Metabolism: An Overview

Glutamine (Gln) is the most abundant amino acid, playing a pleiotropic role in cancer development [1]. The upregulation of Gln metabolism is a hallmark of PCa and several other types of tumors, including glioblastoma (GBM) [2], colorectal [3], pancreatic [4], lung [5], and prostate cancer (PCa) [6]. Gln is a water-soluble amino acid [7], and its cell uptake requires membrane transporters of the solute carrier (SLC) family SLC1, SLC6, SLC7, and SLC38, as reviewed elsewhere [8,9]. Several Gln transporters were shown to regulate tumor cell growth and were associated with PCa progression to castration-resistant and metastatic disease, including ASCT2 (SLC1A5) [10], LAT1 (SLC7A5) [11,12], and LAT2 (SLC7A8) [13]. Recent studies have demonstrated that amino acid transporters possess high plasticity and redundancy, providing metabolic flexibility to tumor cells [14]. Transporters with different substrate specificity and transport mechanisms maintain the intracellular amino acid pool. Gln transporters are classified as uniporters (loaders), providing amino acid accumulation (influx) inside the cells and antiporters (harmonizers), mediating amino acid exchange (influx/efflux) [15,16]. Gln transporters are not only specific for Gln but also mediate the transport of other amino acids [14,16]. Furthermore, cells with the knockout of the ASCT2 (SLC1A5) transporter demonstrated a high adaptation to amino acid imbalance via the upregulation of redundant transporters such as ASCT1 (SLC1A4) and SLC38A2 (SNAT2) [14,17]. 

When transported into the cells, Gln can be catabolized by several enzymes, including glutaminase (GLS), carbamoyl-phosphate synthase (CPS/CAD), and glutamine phosphoribosyl pyrophosphate (PRPP) amidotransferase (GPAT). Gln serves as a nitrogen donor for purine and pyrimidine nucleobases and is catabolized by CAD and GPAT, the enzymes contributing the intermediates for the de novo biosynthesis of pyrimidines [18,19,20]. Gln-derived nitrogen is significantly enriched in metabolites contributing to pyrimidine and purine nucleotides in advanced PCa cells [21]. GLS provides a Gln-derived carbon to fuel the tricarboxylic acid (TCA) cycle. TCA reactions supply precursors for the biosynthetic pathways and provide NADH and flavin adenine dinucleotide (FADH2) for the electron transport chain (ETC) [22,23]. Furthermore, intracellular Gln can be exchanged for other amino acids through Gln antiporters, such as LAT1 (SLC7A5), LAT2 (SLC7A8), and SNAT3 (SLC38A3) [8,24,25,26]. Recent studies have demonstrated that the mitochondrial variant of the SLC1A5 transporter localized in the inner mitochondrial membrane is essential for transporting Gln into the mitochondria and following glutaminolysis [27]. 

At the first step of GLS-driven glutaminolysis, the amidohydrolase enzyme GLS converts Gln into glutamate (Glu), generating ammonia as a byproduct. The human genome includes two genes, GLS encoding two protein isoforms: kidney-type glutaminase KGA and glutaminase C (GAC) [28,29], and the GLS2 gene also encoding two protein isoforms: liver-type glutaminase (LGA) and glutaminase B (GAB) [30]. The experimental and computational analyses of GLS cellular localization yielded controversial results suggesting that GLS isoforms can be localized in the cytoplasm and mitochondria in different cell models, including PCa [31]. Ammonia generated as a byproduct of Gln hydrolysis can be metabolically recycled by glutamate dehydrogenase (GDH), which catalyzes the reductive amination of α-ketoglutarate (α-KG) and the synthesis of Glu, and glutamine synthetase (GS), also known as glutamate-ammonium ligase, catalyzing Gln production [32]. The expression levels of GDH or GS are increased in several types of cancers [32,33,34,35]. GS activity is essential to fuel de novo purine biosynthesis and maintain the growth of GBM tumors, as Gln replenishment from circulation is limited, and Gln is mainly synthesized by tumor cells or supplemented by surrounding astrocytes [36]. The GS-driven de novo nucleotide synthesis enables efficient DNA repair and promotes radiation resistance [37]. Recycling Gln-derived ammonia is critical for the synthesis of other amino acids downstream of Gln, including proline (Pro), alanine (Ala), asparagine (Asn), and aspartate (Asp) [32,38,39]. Asp is produced by glutamic-oxaloacetic transaminase (GOT1 and GOT2) [40] and is necessary for de novo pyrimidine and purine synthesis, playing a critical role in regulating highly proliferating cells [41]. In addition, ammonia buffers the surrounding tumor cells, preventing its high acidification due to glycolytic lactate production. Thus, Gln-derived ammonia can prevent acid stress caused by lactate excretion and improves tumor cell survival [42]. An intratumoral accumulation of ammonia leads to a reduction in T cell function and tumor immune evasion [43]. The blood level of ammonia is a biomarker of several malignancies, including PCa [44,45]. In the liver, ammonia is converted into the non-toxic metabolite urea to be eliminated from the body.

The portion of the mitochondrial Glu is transported into the cytoplasm by transporters of the SLC25 carrier family [46,47]. Four transporter molecules regulate the Glu exchange between cytosol and mitochondria, including SLC25A12, SLC25A13, SLC25A18, and SLC25A22. The pool of cytosolic Glu is also replenished by Glu plasma membrane transporters such as excitatory amino acid transporters (EAATs) [48]. Cytosolic Gln contributes to the production of glutathione (GSH), a key antioxidant that neutralizes reactive oxygen species (ROS) and reactive nitrogen species (RNS) [49,50]. GSH is synthesized in cytosol from Gln, cysteine (Cys), and glycine (Gly) in a two-step ATP-dependent process mediated by the enzymatic activity of glutamate cysteine ligase (GCL), and then GSH synthetase (GSS). The xCT (SLC7A11)/CD98hc (SLC3A2) transporter mediates the uptake of extracellular cystine (Cys-S-S-Cys), a precursor of Cys [51]. A reduced sulfhydryl form of GSH can be converted to the oxidized glutathione disulfide (GSSG) or S-nitrosoglutathione (GSNO) forms under oxidative or nitrosative conditions, respectively [52,53]. A high concentration of GSH was reported for some malignancies [54,55], and its deficiency results in oxidative/nitrosative stress and causes DNA damage, lipid peroxidation, and protein modifications [49,50]. In addition to its antioxidant role, GSH also modifies hundreds of intracellular signaling proteins by S-glutathionylation, the reversible protein post-translational modification leading to the formation of disulfide bonds between cysteine and GSH residues [56]. GSH serves as a reductant to modify the oxidized amino acid residues of different signaling proteins, including transcriptional factors, and therefore regulates redox-dependent signal transduction mechanisms [50].

In radioresistant GBM cells, a bi-directional Gln transporter SLC25A22 is upregulated, and its transporter activity becomes unidirectional from mitochondria to cytosol, leading to the increase in the portion of cytosolic Glu. The cytosolic accumulation of Glu induces GSH synthesis, remodeling of the extracellular matrix, and GMB aggressiveness [57]. 

Cytosolic Glu is catabolized by amidophosphoribosyltransferase (PPAT), contributing to forming the intermediates for de novo purine synthesis [58]. Glu also serves for the biosynthesis of amino acids such as Pro and arginine (Arg) and other critical metabolites [59]. Furthermore, cytosolic Glu also plays several tissue-dependent physiological roles. For example, Glu transport from mitochondria is required for glucose-induced insulin secretion by pancreatic β-cells. Here, cytoplasmic Glu potentially serves as an additional amplifying messenger of the Ca^2+^ signaling for triggering insulin secretion [46]. In the central nervous system, Glu is released by neurons and astrocytes. It plays a role as a neurotransmitter [60] and serves as a precursor of another neurotransmitter γ-aminobutyric acid (GABA) synthesized by glutamate decarboxylation in neurons and β-cells [61]. 

**Figure 1:**
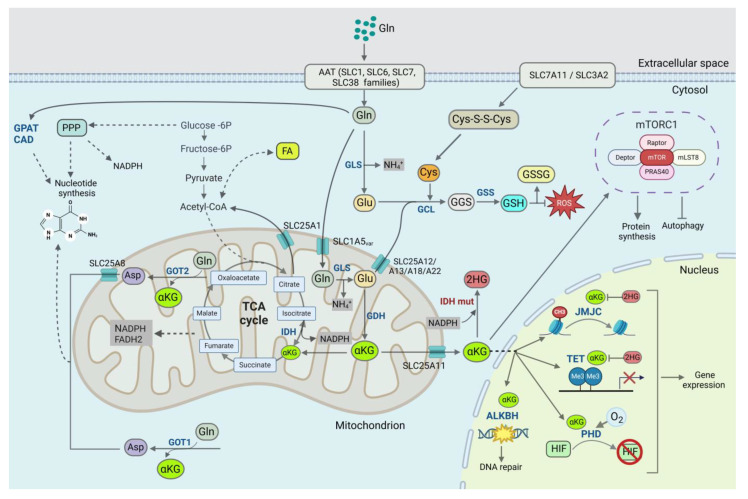
The role of glutamine (Gln) supply in cell metabolism. Gln uptake is mediated by membrane transporters of the solute carrier (SLC) family SLC1, SLC6, SLC7, and SLC38. When transported into the cells, Gln can be catabolized by several enzymes, including glutaminase (GLS), carbamoyl-phosphate synthase (CAD), and glutamine phosphoribosylpyrophosphate (PRPP) amidotransferase (GPAT). Gln is catabolized by CAD and GPAT, the enzymes contributing the intermediates for de novo biosynthesis of pyrimidines. Gln-derived aspartate (Asp) is produced by glutamic-oxaloacetic transaminase (GOT1 and GOT2) and contributes to de novo pyrimidine and purine synthesis. The mitochondrial variant of the SLC1A5 transporter (SLC1A5var) transports Gln into mitochondria. GLS converts Gln into glutamate (Glu), generating ammonia (NH_4_^+^) as a byproduct. The mitochondrial Glu is oxidatively deaminated by glutamate dehydrogenase (GDH) to α-ketoglutarate (α-KG). Gln-derived αKG activates the mammalian target of rapamycin complex 1 (mTORC1). Mitochondrial αKG can enter the mitochondrial tricarboxylic acid (TCA) cycle, serving as a carbon source, or can be transported to the cytosol by the SLC25A11 transporter. A nuclear αKG is a cofactor for the Fe(II)/2-oxoglutarate-dependent dioxygenases, including Jumonji-domain (JMJC)-containing histone demethylases, ten-eleven translocation (TET) DNA demethylases, hypoxia-inducible factors (HIF)-prolyl hydroxylases (PHD), and AlkB homologs (ALKBH) chromatin-modifying enzymes. The enzymes of the isocitrate dehydrogenase (IDH) family are responsible for the reversible conversion of αKG to isocitrate. The αKG production from isocitrate by oxidative decarboxylation is associated with generating reduced nicotinamide adenine dinucleotide phosphate (NADPH), whereas ICT production from αKG by reductive carboxylation requires NADPH oxidation. IDH mutations lead to the NADPH-dependent production of the oncometabolite D-2-hydroxyglutarate (2HG) from αKG. The portion of the mitochondrial Glu is transported into the cytoplasm by transporters of the SLC25 carrier family. Glu is catabolized by amidophosphoribosyltransferase (PPAT), contributing to de novo purine synthesis. Glutathione (GSH), a key antioxidant that neutralizes reactive oxygen species (ROS), is synthesized from Gln, cysteine (Cys), and glycine (Gly) by glutamate cysteine ligase (GCL) and GSH synthetase (GSS). SLC7A11/SLC3A2 transporter mediates the uptake of extracellular cystine (Cys-S-S-Cys), a precursor of Cys. A reduced GSH can be converted to the oxidized glutathione disulfide (GSSG) form. FA: fatty acids; FADH2: flavin adenine dinucleotide; Fructose-6P: fructose 6-phosphate; Glucose-6P: glucose 6-phosphate; mLST8: mTOR-associated protein, LST8 homolog; RPAS40: proline-rich Akt substrate. Created with BioRender.

The mitochondrial Glu is oxidatively deaminated by GDH to α-KG, also known as 2-oxoglutarate (2-OG) [62]. Mitochondrial αKG is also produced by transaminases such as aspartate transaminase (AST/GOT), mediating an amino group transfer between aspartate and glutamate [63]. Mitochondrial αKG can enter the mitochondrial TCA cycle, serving as a source of carbon, or can be transported to the cytosol by SLC25A11 transporter, exchanging αKG for malate (Figure 1) [6,27,63,64]. Gln-derived αKG activates the mammalian target of rapamycin complex 1 (mTORC1) signaling that induces cell growth and inhibits autophagy [6,65]. Reductive carboxylation of αKG is required for its conversion to citrate, which is subsequently utilized for the acetyl-CoA and aspartate production. In turn, acetyl-CoA is a precursor for fatty acid synthesis and a modulator of protein modifications, including epigenetic marks such as histone acetylation [66,67].

Citrate-derived aspartate (Asp) and cytosolic Glu are utilized for the synthesis of purine and pyrimidine nucleotides and amino acids [19,68]. A shortage in Asp and Gln leads to nucleotide deficiency, DNA damage, and the inhibition of cell proliferation [6,36,69]. In addition, αKG is a cofactor for the Fe(II)/2-oxoglutarate-dependent dioxygenases (OGDD), including Jumonji-domain (JMJC)-containing histone demethylases, ten-eleven translocation (TET) DNA demethylases and hypoxia-inducible factors (HIF)-prolyl hydroxylases (PHD) essential for HIFα-hydroxylation and consequent degradation [70]. Consistent with this observation, Gln deprivation causes the epigenetic reprogramming of PCa cells by global changes in histone modifications, e.g., an increase in H3K27me3 methylation levels due to a block in the enzymatic activity of α-KG-dependent OGDD enzymes [6]. αKG-dependent dioxygenases include the family of HIF-prolyl hydroxylases (PHDs). The PHD enzymes are oxygen (O_2_) sensors that mediate the O_2_-dependent hydroxylation of HIF. The activity of PHDs is inhibited by hypoxia and plays a central role in regulating hypoxia signaling during tumor initiation, growth, and metastases [71]. Gln blockage in PCa cells inhibits the activity of the AlkB homologs (ALKBH) family of the αKG-dependent dioxygenases. ALKBH proteins play an essential role in repairing DNA alkylation damage. Gln deficiency sensitizes PCa cells to alkylating agents such as methyl methanesulfonate (MMS), whereas αKG replenishment rescues this effect [72]. Despite the essential role of αKG in regulating chromatin modification and DNA repair, the mechanisms of its transport from cytosol to the nucleus remain poorly understood. Instead, the recent data suggest that some enzymes contributing to αKG synthesis, such as GDH and isocitrate dehydrogenase 2 (IDH2), are redirected to the nucleus, providing on-site αKG production [73,74]. 

The enzymes of the IDH family are responsible for the reversible conversion of αKG to isocitrate (ICT). The αKG synthesis from ICT by oxidative decarboxylation is associated with generating reduced nicotinamide adenine dinucleotide phosphate (NADPH), the most important reducing equivalent, whereas ICT production from αKG by reductive carboxylation requires NADPH oxidation. The gain-of-function mutations of IDH1 and IDH2 genes commonly occur in GBM and acute myeloid leukemia (AML) and are associated with accelerated tumor cell proliferation and inhibited differentiation [75,76]. At the mechanistic level, IDH mutations lead to the NADPH-dependent production of the oncometabolite D-2-hydroxyglutarate (D-2HG) from αKG [77]. The oncogenic role of D-2HG is mainly driven by its inhibition of the αKG-dependent dioxygenases and, consequently, a broad epigenetic resetting typical for IDH-mutated tumors [77]. 

In addition, Gln metabolism regulates the pentose phosphate pathway (PPP), one of the central carbon metabolic pathways splitting from glycolysis at glucose-6-phosphate (G6P) and producing ribose-5 phosphate (R5P), a precursor for ribonucleotide synthesis. Furthermore, PPP is the major pathway to generate NADPH [78], a main reducing agent protecting cells against oxidative stress and driving biosynthetic reactions such as amino acids, fatty acids, and nucleotide synthesis [79]. A high activity of glucose 6-phosphate dehydrogenase (G6PD), the first and rate-limiting enzyme of PPP, is common for different malignancies [80]. Gln metabolism and PPP are tightly interconnected: Gln deprivation activates G6PD, whereas a loss of G6PD function activates glutaminolysis in cancer cells [81,82]. In this way, tumors have redundant protection against oxidative stress. 

The tumor microenvironment tightly regulates Gln metabolism in tumor cells. In particular, under hypoxic conditions, Gln consumption is elevated, and Gln carbon is mainly used for fatty acid production through the reductive carboxylation of αKG and citrate synthesis [83,84,85], whereas the Gln-derived nitrogen is expelled from cells in the form of dihydroorotate to prevent ammonia-induced toxicity [85]. Hypoxia downregulates mitochondrial Gln oxidation and reprograms tumor metabolic pathways by activating the HIF transcriptional program to support cell growth and survival [83,84]. The HIF1α-dependent cell reprogramming induces αKG production, ICT, and 2HG synthesis by the wild-type IDH enzymes [84], inhibition of the alpha-ketoglutarate dehydrogenase (αKGDH) converting αKG to succinyl-CoA [83], and expression of Gln transporters such as SLC38A2 [86] and SLC38A1 [87]. The HIF-2α-dependent expression of the mitochondria-specific SLC1A5 variant induced by hypoxia is important for the Gln-dependent GSH synthesis and ATP production [27]. In addition, HIF-2 induces the expression of Gln transporter SLC7A5 and activation of mTORC1, leading to cell proliferation and tumor growth [88]. 

Thus, cancer cells have a higher demand for Gln to sustain their viability and tumor growth. The products of Gln metabolism are essential for antioxidant protection, transcriptional activity, and epigenetic regulation. Gln metabolism fuels the mitochondrial TCA cycle, serving as a carbon source. Gln contributes to the biosynthesis of proteins and nucleotides as a nitrogen source, enabling tumor cell survival and proliferation.

## 2. The Impact of Gln Metabolism on Tumor Immune Response

Gln is an essential component of the tumor microenvironment regulating different immune cell populations and antitumor immune response [89]. The Gln uptake by tumor cells is essential for inhibiting programmed death-ligand 1 (PD-L1) expression and activating antitumor immunity [90]. The recent studies in melanoma and colon cancer murine syngeneic tumor models demonstrated that tumor cells and immune cell populations such as antigen-presenting conventional dendritic cells (cDC) responsible for the priming of the CD8+ cytotoxic T cells and CD4+ T cells are competing for Gln uptake. Tumor cells outcompete DCs for Gln uptake, thus inhibiting DC functions. The intratumoral Gln supplementation boosts the immune response in these models [91]. 

Remarkably, the inhibition of Gln uptake by Gln antagonist 6-diazo-5-oxo-L-norleucine (DON) and its prodrugs JHU-083 and DRP-104 are reported to improve the antitumor immune response [92,93,94]. DRP-104 boosted the function of CD4+ and CD8+ T cells, decreased T cell exhaustion, reduced T_reg_ population, increased efficacy of anti-PD-1 immunotherapy, and induced immunologic memory in murine models [93,95]. The inhibition of Gln uptake by Gln antagonist prodrug JHU-083 reduced tumor growth and activated antitumor immune responses in the immunocompetent syngeneic models of murine colon cancer. The Gln blockage was associated with the increased population of the highly proliferative activated and long-lived tumor-infiltrating CD8+ T cells. Of importance, the treatment with JHU-083 resulted in the suppression of oxidative phosphorylation (OXPHOS) and glycolysis in tumor cells but upregulated oxidative metabolism in T cells, suggesting a difference in their metabolic response to Gln blockage [92]. Inhibiting Gln flux by SLC1A5, SLC38A2, and SLC7A5 transporters with the V9302 small molecule antagonist boosts the tumor infiltration of CD8+ T cells in syngeneic breast cancer models and sensitizes experimental tumors to the programmed death-1 receptor 1 (PD-1) blockade [96].

In line with these findings, the inhibition of Gln metabolism with V9302 and Bis-2-(5-phenylacetamido-1,3,4-thiadiazol-2-yl)ethyl sulfide (BPTES) in murine syngeneic colorectal carcinoma model increases PD-L1 expression in tumor cells, activates T-cell-mediated Fas cell death signaling, and synergizes with anti-PD-L1 immunotherapy [90]. The V9302-driven mechanisms of immune boost involve the induction of the autophagy-mediated degradation of B7H3 (CD276), an immune checkpoint molecule inhibiting T-cell activation [96]. The Gln metabolism-based gene expression signature is predictive of the tumor response to the immune checkpoint inhibitors in patients with lung adenocarcinoma [97]. Treatment with JHU-083 potentiated the antitumor effect of the EGFR peptide vaccine (EVax) by increasing tumor infiltration of CD8+ T cell and CD4+ Th1 cells and inhibiting immune suppressive cells, including regulatory T cells (Tregs), myeloid-derived suppressor cells (MDSCs), granulocytic myeloid-derived suppressor cells (G-MDSCs), and tumor-promoting CD4+ Th17 cells in the syngeneic lung tumor models carrying human epidermal growth factor receptor EGFR^L858R^ mutation and transgenic EGFR^L858R^-driven murine lung cancer model [98]. In contrast to the suppressive effect on tumor cell growth, Gln blockage in these models induced OXPHOS in antitumor immune cells, including CD8+ T cells and Th1 cells, and led to their expansion. In line with these observations, the treatment of the murine syngeneic PCa and bladder cancer models with the JHU-083 inhibitor led to the reprogramming of the tumor-associated macrophages (TAMs) from an immunosuppressive to inflammatory state and increased TAM proliferation and glycolysis [99]. The oncometabolite D-2HG produced in IDH-mutant tumors from αKG contributes to inhibiting antitumor immunity via the metabolic reprogramming of CD8+ T cells and decreasing their proliferation and antitumor toxicity [100]. 

Of note, the effect of the Gln metabolism inhibition on the immune response is a matter of controversy. For example, GLS inhibition with CB-839 negatively impacted immune activation by the anti-PD1 immunotherapy in the transgenic murine models of KRAS/Serine-threonine kinase 11 (STK11)-mutant lung adenocarcinoma carrying an immune suppressive mutation in the STK11 gene. This effect is associated with a dependency of CD8+ T cell activation on the availability of the GLS product, Glu, in the tumor microenvironment of the STK11-deficient models [101]. This study demonstrated that GLS activity is important for the anti-immune response upon immune stimulation with checkpoint inhibitors. The controversy of the experimental finding about the interaction of the Gln blockage and immune therapy can be explained by the employment of the different in vivo models and distinct mutation profiles of the experimental tumors. The careful analysis of the outcomes of the clinical trials evaluating GLS inhibition in combination with immune checkpoint inhibitors in patients with solid tumors, including non-small-cell lung cancer (NSCLC), melanoma, and clear cell renal cell carcinoma (ccRCC) (NCT03894540, NCT02771626, and NCT04265534 that were all terminated), would be critical for assessment the potential clinical benefit from combining Gln metabolism blockage and immune therapies. 

## 3. The Role of Gln Metabolism in Prostate Cancer Development and Progression

Gln plays a pleiotropic role in prostate tumorigenesis by regulating tumor initiation, progression, metastases, and therapy resistance [102,103]. Gln deprivation inhibits PCa cell proliferation, survival, and tumor-initiating potential, induces the endoplasmic reticulum (ER) stress response, oxidative stress, and DNA damage, and sensitizes PCa cells to radiotherapy [6,104]. The genetic knockdown of Gln transporter ASCT2 (SLC1A5) inhibited PCa proliferation in vitro and the growth of xenograft prostate tumors [105]. Furthermore, Gln utilization in PCa can increase during tumor progression. As a metastatic derivative of the PC3 cell line, PC3M cells are more sensitive to GLS inhibitors CB-839 (telaglenastat) than their less metastatic isogenic counterparts [106]. Activation of the pro-survival autophagy in PCa cells in response to Gln deficiency helps replenish intracellular Glu and αKG levels and makes tumors less sensitive to the inhibition of Gln uptake [6].

Gln metabolism is regulated by oncogenes involved in PCa initiation and progression. The expression levels of Gln transporters ASCT2 (SLC1A5), LAT1 (SLC7A5), and ASCT1 (SLC1A4) are dynamically regulated by several oncogenes driving PCa progression, including MYC, androgen receptor (AR), and mTORC1 [107,108,109]. MYC is commonly upregulated in primary and metastatic PCa [110,111,112] and is associated with poor clinical prognosis [6,113]. A highly activated MYC transcriptional program drives PCa progression toward androgen-independent metastatic disease [113]. The MYC-promoted activation of Gln uptake and glutaminolysis induces oncogene-driven Gln addiction in PCa cells; however, in non-malignant prostate epithelial cells, Gln is dispensable for energy production and biosynthesis [6,103]. By suppressing miR-23a/b, MYC also positively regulates the expression of GLS involved in the first step of Gln catabolism by converting Gln into Glu [6,114]. GLS is upregulated in several types of cancer, including PCa [115,116,117,118]. Pancreatic ductal adenocarcinoma (PDAC) with oncogenic KRAS has an increased αKG production to maintain redox homeostasis and, therefore, possesses GLS dependence [119]. KRAS mutations are found in about 7% of PCa [120], suggesting additional mechanisms of Gln addiction. At the early stage of PCa development, AR drives the expression of the KGA isoform of GLS gene. During PCa progression, tumor cells switch the expression of the GLS gene from the KGA isoform to the more enzymatically active and androgen-independent GAC isoform. This GLS switch contributes to the activation of glutaminolysis and the development of PCa resistant to androgen deprivation therapy [121]. The high expression of the oncogenic drivers, such as MYC and AR, makes PCa particularly dependent on Gln availability. Indeed, Gln is a conditionally essential (semi-essential) amino acid; it is dispensable for normal epithelial prostate cells but becomes essential in PCa. 

Gln can be synthesized endogenously by GS. However, tumor cells often depend on extracellular Gln to meet their metabolic demands. GS, a key enzyme of de novo Gln synthesis, is downregulated in PCa tissues compared to non-malignant prostate epithelia [122]. The inhibited Gln anabolism makes PCa highly dependent on the exogenous source of Gln [21]. In contrast, the Gln anabolic pathway was shown to be upregulated in cancer-associated stroma cells [123,124]. DNA methylation profiling revealed that prostate cancer-associated fibroblasts (CAFs) are characterized by the epigenetic silencing of the RAS inhibitor, the RAS protein activator–like 3 (RASAL3), and therefore possess an activation of oncogenic RAS signaling. A high RAS activity in CAFs promotes micropinocytosis and induces Gln synthesis and secretion. CAF-secreted Gln is taken by tumor cells that highly express the Gln transporters SLC1A5 and SCL38A2 and is utilized to maintain tumor growth [124]. CAFs possess highly activated Gln anabolic pathways and maintain tumor growth under Gln-deprived conditions. The targeting of Gln synthesis in CAFs by inhibition of the GS induced regression of the experimental ovarian tumors [123]. Furthermore, CAFs could also have a downregulation of p62, c-MYC, and low mTORC1 activation, leading to decreased metabolic detoxification. As a result, CAFs release ROS and GSSG, triggering the anti-oxidative response and increasing the reduced GSH in tumor cells [125,126]. CAF-derived exosomes induce metabolic reprogramming in prostate cancer cells and increase the Gln contribution to the TCA cycle metabolites [127]. These studies highlight the critical role of the microenvironment as a regulator of Gln metabolism in PCa. 

Prostate tumors have a hierarchical organization where cancer stem cell (CSC) populations maintain and propagate tumors [103]. CSCs and their non-tumorigenic progenies within the same clone can share a common genotype but display different epigenetic profiles, resulting in changes in multiple signaling pathways. Many of these pathways confer cell adaptation to microenvironmental stresses, including hypoxia, nutrient shortage, and anti-cancer therapies [128]. Furthermore, some CSC populations are relatively radioresistant due to high DNA repair capability, activation of the cell survival pathways, low proliferation, and metabolic adaptations [102,129,130,131]. Glutaminolysis is essential for maintaining CSC populations. Inhibition of glutaminolysis by Gln depletion, by chemical inhibition of the critical regulators of Gln metabolism such as GLS and MYC, or genetic knockdown of these genes and Gln transporters resulted in the inhibition of the essential CSC-driving signaling mechanisms (e.g., WNT/β-catenin; oxidative stress response; DNA damage response), CSC depletion in vitro and in vivo, and tumor radiosensitization [6]. Aldehyde dehydrogenase positive (ALDH^+^) PCa CSC populations possess radioresistant, tumor-initiating, and metastatic properties [130,132,133] and have a high intracellular αKG level compared to the ALDH-negative (ALDH^-^) counterparts [6]. The intracellular αKG pool in ALDH^+^ cells is depleted in response to Gln starvation, suggesting that Gln blockage is an efficient way to radiosensitize and eradicate ALDH^+^ PCa stem cells [6]. 

Consistent with the findings for PCa models [6], analysis of other types of tumors also confirmed that Gln availability is essential for tumors to withstand radiation therapy. In particular, human GBM and nasopharyngeal carcinoma cells with acquired radioresistance have high intracellular levels of Gln and increased activity of GS, necessary for nucleotide synthesis and efficient DNA repair [37]. A study conducted on neuroblastoma cells revealed that Gln depletion results in increased radiosensitivity in non-MYCN amplified cell lines. In contrast, MYCN-amplified cell lines exhibited radioresistance under the same conditions, suggesting that targeting Gln metabolism can be beneficial for the treatment of c-MYC-driven but not MYCN-amplified neuroblastoma [134]. Of note, in vitro analyses demonstrated that targeting Gln metabolism activated the pro-survival ATG5-mediated autophagy, replenishing cell energy and biosynthesis under nutrient-deprivation conditions, and its blockade resulted in more efficient radiosensitization of PCa cells [6,135]. Furthermore, Gln metabolism plays a role in PCa chemoresistance. Gln deprivation and GLS inhibition, achieved by genetic knockdown or chemical drug CB-839, reduced cell proliferation and ATP production in docetaxel-resistant and sensitive PCa cells [69]. 

Gln metabolism serves as a source of prognostic biomarkers in PCa. Plasma level of Gln was correlated with a short prostate-specific antigen (PSA) doubling time (PSA-DT) and can be used for identifying patients at risk of disease progression [6]. Serum Glu level was also found to be higher in patients with PCa compared to healthy donors [136]. Immunohistological examination of the GLS protein expression in human prostatic specimens confirmed that it is highly expressed in PCa compared to benign prostate hyperplasia (BPH) tissues [69,118]. High levels of GLS protein expression were correlated with worse overall survival in PCa patients after the transurethral resection of the prostate (TURP) [69]. A high MYC and GLS gene expression was significantly associated with shorter PSA relapse-free survival in patients with PCa treated with radiotherapy [6]. 

These findings indicate that the Gln metabolic pathways are vital in regulating tumor initiation, progression, and therapy resistance, providing fertile ground for developing prognostic biomarkers and therapeutic targets. Table 1 summarizes the selected key findings describing the role of glutamine metabolism and its regulation in prostate cancer cells.

## 4. Targeting Glutamine Metabolism as a Strategy to Enhance Therapy Outcomes in Cancer

The features of the altered metabolism of malignantly transformed cells have long been the focus of translational research. One area of interest has been to create a deficit of specific amino acids to treat auxotrophic tumors and, consequently, study the effects of such deprivation [145]. Among the various amino acids, Gln has received particular attention due to its significant role in many reactions of cell metabolism as described above. Accumulating evidence suggests that the therapeutic targeting of Gln metabolism brings promise to increase the efficacy of conventional treatments [146].

**Figure 2:**
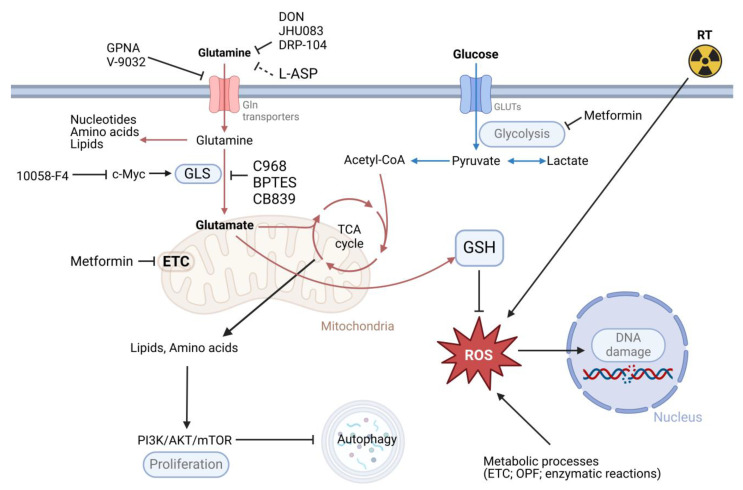
Inhibition of Gln metabolism and the effects of possible combination treatments. BPTES: bis-2-(5-phenylacetamido-1,3,4-thiadiazol-2-yl)ethyl sulfide; DON: 6-Diazo-5-oxo-L-norleucine; C968: compound 968; Gln: glutamine; Glu: glutamate; GLS: glutaminase; GLUTs: glucose transporters; GPNA: L-γ-Glutamyl-p-nitroanilide; GSH: glutathione; L-ASP: L-asparaginase; mTOR: mammalian target of rapamycin; ROS: reactive oxygen species; TCA cycle: tricarboxylic acid cycle; ETC: electron transport chain; OPF: oxidative protein folding; PI3K: phosphoinositide 3-kinases; RT: radiotherapy. Created with BioRender.

Gln-depleting drugs have been developed for over 50 years (Figure 2), but many of them have shown toxicity toward normal tissues during preclinical studies. For instance, L-Gln antagonists like acivicin, 6-Diazo-5-oxo-L-norleucine (DON), and azaserine caused significant gastrointestinal side effects and neurotoxicity. The inhibitors of intracellular Gln transport, such as L-γ-Glutamyl-p-nitroanilide (GPNA) and its derivative V-9032, were ineffective in reducing Gln concentration and are characterized by a lack of specificity [147,148,149]. 

The Gln antagonist DON has been known as a promising anticancer drug since the middle of the last century [150]. Although DON has demonstrated promising anti-tumor efficacy in clinical studies, its further clinical translation was halted by dose-limiting toxicities. DON is a broad metabolic inhibitor blocking the activity of several Gln-utilizing enzymes in tumor and normal tissues. In contrast, the recently described DON peptide prodrug called DRP-104 is preferentially converted to DON in tumor tissues, minimizing normal tissue toxicity. Inhibition of Gln utilization with DON and its prodrugs JHU083 and DRP-104 boost the anti-tumor immune response [92,93,94], suggesting the rationale of their combination with immune checkpoint inhibitors. Recent studies demonstrated that DRP-104 blocked both carbon and nitrogen Gln pathways in castration-resistant PCa (CRPC) cells and inhibited their growth in vitro and in vivo [151]. DRP-104 has been tested in clinical trial NCT04471415 to assess the preliminary safety and efficacy in patients with advanced solid tumors as a single drug or combined with anti-PD-L1 immunotherapy (Atezolizumab). The study was recently terminated, and no results have yet been posted. 

Compared to the inhibitors of Gln uptake, GLS inhibitors, such as C968 [152], bis-2-(5-phenylacetamido-1,3,4-thiadiazol-2-yl)ethyl sulfide (BPTES) [153], and CB-839 (telaglenastat) [154], have been more translationally advanced drugs. They have proven effective as a monotherapy and in combination with chemotherapy in preclinical studies and several clinical trials. Increasing evidence suggests that GLS contributes to the growth and malignancy of certain tumors. 

As a key regulator of Gln catabolism, GLS contributes to GSH synthesis and plays an essential role in the response to oxidative stress. The excessive production of ROS, which leads to the misbalance between ROS and antioxidant cell capacity and, consequently, to DNA damage, is the main mechanism of the radiotherapy-mediated tumor cure. Recent findings have suggested that GLS is involved in acquiring radioresistance, making GLS inhibitors a promising strategy to improve the outcomes of cancer radiotherapy. Inhibition of GLS with BPTES or genetic silencing of GLS or GLS2 increased the radiation sensitivity of lung and prostate tumor cell lines [6,155,156]. 

The most investigated and clinically advanced GLS inhibitor is CB-839, a derivative of BPTES, also known as telaglenastat. In preclinical studies, CB-839 has demonstrated cytotoxic effects on various types of tumors, including triple-negative breast cancer. In vitro and in vivo analyses using xenograft models have shown its anti-tumor effectiveness in combination with paclitaxel [154]. A study combining CB-839 treatment and radiotherapy demonstrated that GLS inhibition increased the radiation sensitivity of lung cancer cells and mouse xenografts by up to 30%, confirming a tight connection between GLS activity and anti-oxidative tumor capacity [157]. A combination of CB-839 and radiotherapy improved the radiosensitivity of xenograft cervical tumors [158]. The cervical cancer cells with phosphoinositide 3-kinases (PI3K)-activated signaling pathway possess increased sensitivity to Gln blockage and show an activation of the oxidative stress and inhibition of the TCA cycle in response to the treatment with CB-839. These findings indicate that activation mutations in PI3K pathways may serve as a predictive biomarker for sensitivity to CB-839 treatment [158]. 

Another potential genetic marker of tumor sensitivity for GLS inhibition is mutations in IDH genes. The IDH1 mutations are common in GBM and AML and are associated with the NADPH-dependent synthesis of 2-HG from αKG [75,76]. The inhibited GSH biosynthesis in these types of tumors provides metabolic vulnerabilities for IDH1-mutated cancer cells. Of importance, GLS inhibition and IDH1 mutations are synthetically lethal under oxidative stress, and GLS inhibition with CB-839 has been shown to selectively radiosensitize the IDH1 mutated xenograft GBM tumors [159]. A phase Ib clinical trial NCT03528642 examined CB-839/telaglenastat safety and anti-tumor activity in combination with radiation therapy and temozolomide in patients with IDH-mutated diffuse or anaplastic astrocytoma. 

The treatment with CB-839 has been shown to increase radiosensitivity in head and neck squamous cell carcinoma (HNSCC) in vitro and in xenograft models by inducing oxidative stress and DNA damage [160]. Of note, no toxic effect was demonstrated for non-malignant cells, such as cervix epithelial cells, in response to the treatment with CB-839 in combination with different doses of radiotherapy [158]. In line with this study, analysis of the PCa in vitro models demonstrated that patient-derived cell cultures and established cell lines can be radiosensitized by GLS inhibition with CB-839 or MYC inhibition with 10058-F4. In contrast, this treatment was not toxic for BPH cultures and was associated with significantly increased radioresistance in BPH cells [6]. These findings can potentially indicate a reduced risk of normal tissue toxicity for future clinical translation. 

One of the biggest challenges to developing an efficient metabolic inhibitor targeting Gln catabolism is delineating the metabolic fate of Gln in vivo. Recent studies have developed a hyperpolarized [5-^13^C,4,4-^2^H_2_,5-^15^N]-L-Gln compound that enables monitoring of the Gln/Glu conversion in murine xenograft models using hyperpolarized magnetic resonance imaging [161]. This approach confirmed the in vivo on-target effect of the CB-839 inhibitor. 

Following promising preclinical results demonstrating a high antitumor efficacy of CB-839/telaglenastat, this drug has already been tested in several phase II clinical trials for combination with conventional and targeted therapies. For example, a phase II clinical study NCT05521997 has analyzed whether combining the standard cisplatin-based radio-chemotherapy with CB-839/telaglenastat improves progression-free survival (PFS) in patients with advanced cervical cancer compared to radiotherapy alone. A phase II randomized clinical trial ENTRATA (NCT03163667) analyzed CB-839/telaglenastat in combination with mTOR inhibitor everolimus in advanced renal cell carcinoma (RCC) and demonstrated an improvement in PFS in patients treated with combination therapy compared to the patients treated with placebo plus everolimus [162]. The amino acid deprivation, including Gln blockage, inhibits signaling mediated by mTORC1 as a nutrient sensor that could explain a synergistic effect of the therapies targeting Gln catabolism and inhibiting mTOR activity. In contrast, the combination of CB-839/telaglenastat with multiple tyrosine kinase receptor inhibitor cabozantinib did not improve the efficacy of cabozantinib in patients with advanced RCC in the CANTATA phase II randomized clinical trial (NCT03428217), although it was well tolerated [163]. Of importance, a current phase II clinical trial NCT03872427 is analyzing the therapeutic response of the advanced solid tumors to CB-839/telaglenastat depending on the mutations in genes regulating the antioxidant system such as neurofibromatosis-1 gene (NF1), Kelch-like ECH-associated protein 1 KEAP1, nuclear factor erythroid 2-related factor 2 (NRF2), and serine/threonine kinase 11 (STK11). 

Thus, accumulating evidence suggests that CB-839 is a promising anti-cancer drug whose tumor-suppressing activity is attributed to inhibiting the carbon branch of Gln metabolism. Nevertheless, a recent study demonstrated a reciprocal regulation of the carbon and nitrogen pathways of Gln metabolism, contributing to the TCA cycle and synthesis of amino acids and nucleotides, respectively. This study suggested that combining GLS inhibition with CAD inhibition regulating de novo pyrimidine nucleotide synthesis is more efficient in inhibiting xenograft tumor growth than each monotherapy alone [21]. 

The dependence of PCa on Gln catabolism can also be increased by metformin, a drug being used to treat type II diabetes. In PCa cells, metformin inhibits the entry of glucose carbon into the TCA cycle. Instead, PCa cells become dependent on Gln reductive carboxylation for citrate synthesis. Inhibiting GLS-driven Gln catabolism by CB-839 or BPTES makes PCa cells highly sensitive to metformin [137]. In line with this observation, a combination of Gln depletion and metformin treatment led to significantly higher radiosensitization of the autophagy-deficient PCa cells than monotherapy [6]. These findings suggest that the effect of the Gln metabolism inhibition may synergize with metformin in PCa cells. 

Furthermore, the preclinical study suggested that Gln bioavailability in PCa cells can be targeted with bacterial L-asparaginase (L-ASP), which has been used extensively to treat leukemia patients sensitive to asparagine depletion. A treatment with L-ASP led to cell cycle arrest, cell death, and a significant increase in PCa sensitivity to irradiation in vitro and in xenograft models [104]. Notably, PCa-associated fibroblasts were reported as a source of Gln, which supports tumor progression. Thus, Gln-free media did not affect the growth of PCa cells co-cultured with CAF. However, the L-ASP treatment of the PCa–CAF co-culture significantly reduced PCa cell viability [104].

In conclusion, Gln metabolism is a critical factor in tumor response to the oxidative stress caused by radiation and certain types of chemotherapy. As a result, targeting Gln metabolism has emerged as a promising strategy to increase the efficacy of the standard type of treatment. The use of GLS inhibitors, particularly CB-839/telaglenastat, has shown promising safety profile and clinical efficacy in clinical trials. However, despite these successes, there is an urgent need for a better understanding of the role of Gln in tumor metabolism and therapy resistance depending on the genetic profile of cancer cells and the dynamically changing tumor microenvironment. 

## 5. Clinical Trials Targeting Gln Metabolism in Prostate Cancer

Gln metabolism has emerged as a critical area of interest in cancer research. It is driven by the understanding that cancer cells exhibit altered metabolic processes to support their rapid growth and survival. Among these metabolic alterations, the dependency on the non-essential amino acid Gln stands out as a hallmark of cancer metabolism, often referred to as “glutamine addiction” [144,164,165]. Given its essential role in tumor metabolism, Gln has been targeted in numerous therapeutic efforts [146,165]. Different approaches have been tested to reduce prostate tumor growth by targeting the Gln metabolism. The Gln metabolism in tumors depends strongly on the activity of GLS, and therefore, targeting GLS with selective glutaminase inhibitors showed promising results in preclinical studies [166]. 

The GLS inhibitor CB-839/telaglenastat was tested in several clinical trials in solid tumors [167,168,169]. CB-839 was very well tolerated in heavily pretreated patients, with mostly mild (grade 1 or 2) adverse effects such as nausea, fatigue, photophobia, mild liver function elevation, diarrhea, and rash [170]. Despite promising preclinical results, single-drug trials faced challenges in demonstrating significant clinical benefits, particularly in PCa patients. Drug combination studies of CB-839 and Talazoparib (NCT03875313) have been terminated due to challenges in demonstrating significant clinical benefits and slow recruitment. It is important to note that no clinical trials utilizing CB-839 were guided by biomarkers. Preclinical studies have suggested that patients with high GLS expression may benefit from CB-839, as GLS expression is associated with tumor dependence on glutamine [6,69].

Another approach involved the Gln antagonist 6-diazo-5-oxo-L-norleucine (DON), one of the oldest drugs targeting Gln-utilizing enzymes, investigated for years as a potential anticancer agent [171]. Clinical phase I/II studies in the 1950s indicated DON’s antitumor activity [150]. However, despite its potential, severe side effects led to the discontinuation of the trials. To enhance DON’s therapeutic index, the prodrug JHU083 was developed, showing promising results due to reduced toxicity [94,172]. Current efforts are focused on exploring the drug’s safety profile to continue clinical studies. 

Another DON prodrug, DRP-104, demonstrated promising preclinical activity in PCa [151] and has already been enrolled for Phase 1/2 in adult patients with advanced solid tumors (NCT04471415; the study was terminated, and no results posted) [173] and in patients with fibrolamellar carcinoma (NCT06027086; the study has not yet started recruiting participants). 

None of the clinical trials targeting Gln metabolism have shown definitive clinical benefits, indicating the complexity of targeting tumor metabolism. Ongoing research focuses on refining these approaches, improving selectivity, and combining therapies to overcome resistance and enhance efficacy. 

The recent finding identified the pan-cancer gene expression signature predicting tumor co-dependency on GLS and gamma-glutamylcysteine synthetase (gamma-GCS) catalyzes the first step of GSH biosynthesis [174]. This signature is designed to identify the patients most likely to benefit from both GLS targeting and redox balance perturbation. These findings and other studies have provided a rationale for combining GLS inhibition with other targeted drugs and conventional therapies inducing oxidative stress, such as radiotherapy [6]. 

The continued exploration of Gln metabolism is crucial for developing targeted cancer treatments and improving patient outcomes.

## 6. The Interplay between Glutamine Metabolism and Androgen Signaling

**Figure 3:**
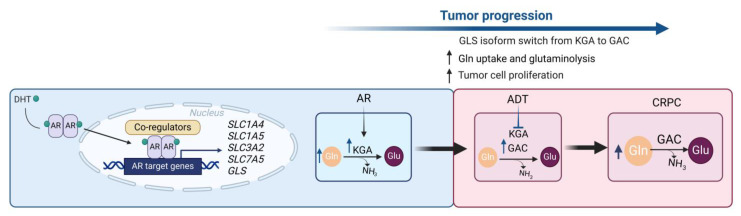
An interplay between androgen signaling and Gln metabolism during prostate cancer progression. The AR plays a pivotal role in the progression of PCa by orchestrating and regulating the Gln metabolic network and influencing the GLS isoform switch. The transition to CRPC prompts a substantial shift towards glutaminolysis to meet the increased energy demands of PCa cells. AR: androgen receptor; DHT: 5α-dihydrotestosterone; Gln: glutamine; Glu: glutamate; CRPC: castration-resistant prostate cancer; GAC: glutaminase C; GLS: glutaminase; KGA: kidney-type glutaminase. Created with BioRender.

The AR is a ligand-dependent nuclear receptor and a crucial player in the development and progression of PCa, influencing differentiation, cell proliferation, apoptosis, and DNA repair mechanisms [144,175,176,177,178]. Moreover, several studies have indicated that the AR regulates cellular metabolism in PCa, including glycolysis, TCA cycle, OXPHOS, and lipid metabolism [121,179]. The AR also directly impacts Gln metabolism by regulating the expression of different key players of Gln utilization (Figure 3). 

Thereby, the AR increases the Gln uptake by modulating the expression of several Gln transporters like *SLC1A4* (ASCT1), *SLC1A5* (ASCT2), *SLC3A2* (4F2hc), *SLC7A5* (LAT1), and *SLC43A1* (LAT3) [10,108,180]. Moreover, the receptor controls the expression of the AR-dependent KGA isoform of GLS, resulting in increased TCA cycle metabolite α-ketoglutarate, a key intermediate of glutaminolysis [108,121]. The influence of the AR on Gln uptake and glutaminolysis, coupled with the imperative role of Gln in achieving peak androgen-mediated PCa, supports the inference that the AR facilitates Gln utilization required for optimal PCa cell growth [10,108,121]. Due to its multifaceted and indispensable role in PCa, AR represents the linchpin of contemporary metastatic PCa therapies [181]. 

The foundation of AR-directed therapy lies in either the withdrawal of the natural ligand, the so-called androgens, or the direct deactivation of the AR by so-called antiandrogens (e.g., apalutamide, enzalutamide, or darolutamide) [181,182]. Consequently, the androgen-induced PCa progression is inhibited, leading to a reduction in tumor size. Following AR activity inhibition, a noticeable reduction in the metabolites stemming from various metabolic pathways is observed [121]. These metabolic pathways include glycolysis, TCA, the pentose phosphate pathway, and glutaminolysis. This decrease in metabolism leads to a shift toward increased intracellular Gln synthesis and intracellular accumulation of intracellular Gln, as glutaminolysis no longer processes it [121]. 

Despite the initial positive treatment response, therapy resistance eventually emerges, and the highly aggressive and fast-growing CRPC develops, representing the biggest challenge in metastatic PCa treatment. During CRPC development, the tumor undergoes several adaptions counteracting supraphysiological androgen levels, including increased androgen synthesis, AR amplification, AR mutation, AR alternative splicing, MYC overexpression, and hyperactive STAT signaling [113,178,183,184]. Moreover, the AR-targeted therapy-induced PCa adaptions provoke metabolic changes, switching the metabolic needs in CRPC [39,144,185,186]. 

This metabolic reprogramming increases Gln utilization in CRPC [121,141,187]. Following the progression of the disease to CRPC or under hormonal therapy inhibiting AR function, the GLS isoform switch occurs in favor of the GAC isoform, allowing advanced PCa cells to favor Gln over glucose to sustain the TCA cycle and augmented proliferation [121]. The elevated Gln metabolism in CRPC cells manifests as increased uptake of Gln and upregulated gene expression concerning critical regulators of glutaminolysis [118,121,188]. This increase in Gln demand is reflected by the overexpression of Gln transporters, such as ASCT2 and LAT1, as well as in the increased expression of the androgen-independent GLS isoform, GAC [12,105,121,180]. Therefore, the treatment-naïve PCa uses the accumulating Gln during androgen withdrawal to balance the therapy-induced energy deficit by adapting to glutaminolysis for PCa progress to CRPC. Furthermore, PCa cells and prostate CSCs exhibit Gln addiction as PCa advances to more aggressive stages [6,135]. Thus, targeting the Gln metabolic network as an Achilles’ heel of PCa offers unique opportunities to tackle therapy resistance and sensitize tumor cells to anticancer treatments and might be more advantageous than targeting AR alone [6,10,12,21,105,108,121,135,137,142,180].

Thus, AR plays a central role in PCa progression by regulating the Gln metabolic network. While AR-targeted therapies initially show promise, the emergence of CRPC brings about adaptive changes, including elevated Gln utilization. The transition to CRPC leads to a shift towards glutaminolysis to meet the energy demands of advanced PCa cells. Gln serves as a central hub in the advanced PCa metabolic network. Targeting Gln metabolism is a promising strategy, as PCa cells, including CSCs, display Gln addiction during disease progression. A comprehensive approach targeting AR and Gln metabolism may offer enhanced efficacy in fighting advanced PCa and overcoming therapy resistance.

## 7. The Impact of Gln Metabolism on Tumor Chemotherapy Resistance

In addition to AR-targeted therapy, representing the medical treatment backbone of metastasized PCa, treatment with taxane-based chemotherapy has been established as a conventional approach for managing metastatic PCa [181]. Docetaxel, a tubulin-binding agent, has been established as the first-line therapeutic choice for metastasized hormone-sensitive and castration-resistant PCa, significantly improving progression-free survival and overall survival [181,189,190,191]. In addition, cabazitaxel has been approved for CRPC patients whose disease has progressed following prior docetaxel treatment [181,192]. Nevertheless, despite the initial taxan-mediated treatment success, nearly all patients become refractory and develop taxan resistance [193]. Due to its pleiotropic role in cell biology, Gln has been linked to chemotherapy resistance in several tumor entities [194]. The involvement of Gln in cisplatin, doxorubicin, gemcitabine, and sorafenib resistance has been reported, whereas a direct connection to Gln in taxane resistance in PCa has not been described yet [194,195]. However, based on findings from other tumor entities, several identified features of docetaxel-resistant PCa cells can be linked to Gln metabolism. 

Several reports have revealed that docetaxel-resistant mCRPC harbors a mesenchymal cell population with increased invasion and motility properties and tumor-forming potential [196,197,198,199,200,201]. These features need specific energy requirements, resulting in a metabolism reprogramming characterized by elevated OXPHOS, GSH production, and ROS scavenging [138,202]. These attributes are linked to Gln metabolism [144]. 

Next to the changed phenotype of docetaxel-resistant cells, multiple molecular resistance mechanisms have been uncovered, including enhanced drug efflux, anti-apoptotic processes, and pro-survival pathways [203,204]. The anti-apoptotic protein Bcl-2 is inactivated by taxane treatment, abolishing Bcl-2′s anti-apoptotic effects. Bcl-2 is overexpressed in docetaxel-resistant cells, thereby counteracting docetaxel-induced apoptosis [203,205,206]. Bcl-2 overexpression is associated with high GSH levels, and the inhibition of GSH synthesis reverses Bcl-2-mediated cisplatin resistance in breast cancer cells [207]. Therefore, alterations in Gln metabolism during the development of docetaxel resistance might increase GSH levels, and contribute to Bcl-2-mediated resistance. 

The ATP-binding cassette sub-family B member 1 (ABCB1) is a membrane-standing ATP-dependent efflux pump defense mechanism against harmful substances, mediating docetaxel resistance by limiting intracellular drug concentrations [208,209]. Drug efflux by ABCB1 in cisplatin-resistant hepatocellular carcinoma has been reported to be reliant on mitochondrial ATP fueled by Gln [195]. Therefore, increased glutaminolysis may confer a survival advantage to docetaxel-resistant PCa cells by facilitating ABCB1-mediated docetaxel efflux.

Drawing from insights from other tumor entities, the molecular mechanisms of docetaxel resistance in PCa can be partially elucidated. However, further research is warranted to substantiate the involvement of Gln metabolism in docetaxel resistance.

## 8. Conclusions

Gln is a non-essential amino acid that plays a pleiotropic role in the development of different types of tumors, including PCa. Gln is dispensable for normal epithelial prostate cells but becomes essential in PCa. A high addiction of PCa cells to the exogenous source of Gln is driven by low expression levels of GS, a key enzyme for de novo Gln synthesis. Furthermore, the dependence of PCa on Gln catabolism can be increased by the mutation in key metabolic regulators such as IDH1 or by treatment with metabolic inhibitors such as metformin or L-ASP. Gln metabolism is tightly regulated by oncogenes involved in PCa initiation and progression, including MYC, AR, and mTOR. The activation of the Gln metabolic pathways contributes to the radio- and chemoresistance and PCa progression to the castration resistance stage. Gln deprivation inhibited PCa growth, survival, tumor-initiating potential, and induced therapy sensitivity. The high importance of Gln metabolism for PCa initiation, progression, and therapy resistance makes it an attractive target for the development of more efficient antitumor strategies. Several clinical trials are currently evaluating the safety and clinical efficacy of targeting Gln catabolism in combination with conventional and targeted therapies in patients with different types of solid tumors, including PCa. A better understanding of the dynamic interplay between tumor cells and the microenvironment in regulating tumor metabolic vulnerabilities and a better understanding of the effect of Gln blockage on the tumor micromilieu might lay the ground for the clinical translation of the Gln inhibitors.

## Figures and Tables

**Table 1:** Selected findings describing the role of glutamine metabolism and its regulation in prostate cancer cells (in chronological order).

No.	Genes/Pathways	Models	Selected Key Findings Related to the Topic	Refs.
1	GLS, MYC, miR-23a/b, glutaminolysis	PCa PC3 cells and lymphoma cells	1. MYC upregulates GLS expression by transcriptional repression of miR-23a and miR-23b; 2. Glutamine withdrawal or GLS knockdown reduces ATP and GSH levels, increases ROS, and inhibits cell proliferation.	[114]
2	GLS (GAC and KGA isoforms)	PCa cell lines PC3 and DU145; breast and lung cancer cell lines	1. GAC but not KGA is localized in mitochondria; 2. The catalytic efficiency of GAC depends on the presence of inorganic phosphate.	[29]
3	MYC, POX/PRODH, interconnection of Gln/Pro metabolic pathways	PCa PC3 cells and lymphoma cells	1. MYC suppresses POX/PRODH expression by upregulating miR-23b* (miR-23b [114] and miR-23b* are processed from the same transcript but differently regulated). 2. MYC induces Pro biosynthesis from Gln.	[39]
4	Glutamine and glucose mediated anaplerosis, mitochondrial complex I	PCa cell lines LNCaP and PC3; the transgenic adenocarcinoma of the mouse prostate (TRAMP) model	Metformin therapy enhances glutamine anaplerosis and synergizes with glutamine metabolism inhibition in cancer cells.	[137]
5	TXNIP, GLS, glucose uptake, glutaminolysis	PC3 cells, BPH, and PCa patient tissue samples	1. GLS is highly expressed in PCa tissues compared to BPH; GLS levels correlate with Gleason scores and TNM stages in patients with PCa; 2. Gln and GLS positively regulate glucose uptake by inhibiting TXNIP expression.	[118]
6	ASTC2-mediated Gln uptake, mTORC1 pathway, E2F-regulated cell cycle genes, FA synthesis	PCa cell lines LNCaP, PC3 and DU145; PC3 xenograft murine model; PCa patient tissue samples	1. ASCT2 expression level is increased in PCa tissues; 2. ASCT2 expression is AR-dependent; 3. ASCT2 mediates Gln uptake in PCa cells; 3. Chemical inhibition of ASCT2 decreases basal OCR and FA synthesis; 4. ASCT2 expression is essential for tumor cell growth in vitro and in vivo.	[10]
7	MYC, miR-205, oxidative metabolism	PCa cells PC3 and docetaxel-resistant PC3-DR cells; CAFs	Docetaxel-resistant PCa cells acquire Gln addiction	[138]
8	MYC, AR, and mTORC1 signaling pathways, SLC1A4, SLC1A5	PCa cell lines LNCaP and VCaP	MYC, AR and mTORC1 oncogenic pathways regulates the expression levels of Gln transporters.	[108]
9	ALKBH, DNA-repair and apoptotic pathways	PC3 cells; MEF; multiple cancer cell lines; PC3 xenograft murine model	1. Gln metabolism regulates the DNA alkylation damage repair by regulation of the α-KG-dependent ALKBH; 2. Combination of DON or CB-839 and alkylating agent MMS inhibits tumor growth in vivo.	[72]
10	Gln and glucose catabolism, mTORC1 pathway, AMPK, GLS (GAC and KGA isoforms), GLS2	PCa cell lines PC3, PC3M, and non-transformed cells RWPE-2 and RWPE-1	Metastatic PCa cells have increased Gln utilization and high sensitivity to GLS and mTORC1 inhibition.	[106]
11	GLS, WNT/β-catenin, cell cycle and apoptosis pathways	PCa cell lines 22Rv1, DU145, PC-3, LNCaP, and non-transformed RWPE-1 cells	GLS knockdown suppresses WNT/β-catenin pathway, inhibits cell proliferation, and induces apoptosis and cell cycle arrest.	[139]
12	RAS, RASAL3, TCA cycle, mitochondrial bioenergetics, PCa neuroendocrine differentiation signaling	PCa cell lines 22Rv1, C4-2B; prostatic fibroblasts derived from the patients with PCa and from murine prostates; BPH cells	1. Epigenetic silencing of the RASAL3 gene in human prostatic CAFs results in oncogenic RAS activity and Gln synthesis and secretion; 2. CAF-derived Gln is utilized by PCa cells and induces neuroendocrine differentiation; 3. ADT promotes epigenetic silencing of RASAL3 in CAFs; 4. A high level of Gln in the blood of the patients with PCa treated with ADT correlates with therapy resistance.	[124]
13	PDHA1, PTEN, lipogenic genes and metabolic pathways	PCa cell lines 22Rv1, LNCaP, PC3, and DU145, PNT2C2; 22Rv1 xenograft murine model; transgenic murine models for prostate-specific deletion of PTEN and PDHA1	1. Gln plays an important role in de novo lipogenesis; 2. Knockdown of PDHA1 decreases the incorporation of Gln and glucose carbon into lipids and cholesterol; 3. PTEN-negative prostate cells have increased Gln carbon incorporation into citrate, fumarate, and malate compared to normal epithelial cells.	[140]
14	Glutaminolysis, TCA cycle, DNA damage signaling, autophagy, MYC, GLS, CSC regulation	PCa cell lines DU145, LNCaP, PC3 and their radioresistant (RR) derivatives; patient-derived cell cultures of PCa and BPH; LNCaP and DU145 xenograft murine models; blood plasma samples of PCa patients	1. Inhibition of Gln metabolism increases oxidative stress, DNA damage and PCa sensitivity to radiotherapy; 2. Activation of ATG-mediated autophagy abrogates the radiosensitizing effect of Gln metabolism inhibition; 3. Gln metabolism regulates CSC populations by the α-KG-dependent epigenetic reprogramming; 4. A high expression of MYC and GLS genes and a high blood level of Gln correlate with a poor prognosis in PCa patients treated with radiotherapy.	[6]
15	Glutaminolysis, TCA cycle	PCa cell lines LNCaP and PC3; the transgenic adenocarcinoma of the mouse prostate (TRAMP) model	1. CRPC cells possess Gln addiction; 2. The upregulation of glutaminolysis and Gln anaplerosis into the TCA cycle are characteristic of castration-resistant PCa.	[141]
16	ASCT2, glutaminolysis, glycolytic and lipid metabolic pathways	PCa cell lines DU145, LNCaP, and PC3; rat model	1. CRPC cells are more Gln dependent than androgen-sensitive PCa cells; 2. DHT induce GLS and ASCT2 expression in androgen-sensitive PCa cells; 3. Inhibition of GLS with BPTES decreased PCa migration and induce cell death; 4. Anti-androgen treatment increases PCa cell sensitivity to GLS inhibition with BPTES; 5. GLS inhibition with BPTES affects glycolytic and lipid metabolism in a cell line-dependent manner.	[142]
17	Gln carbon and nitrogen metabolic pathways, pyrimidine synthesis, CAD, GLS, PI3K-AKT-mTOR pathway	PCa cell lines DU145, LNCaP, PC3, C4-2, C4-2MDVR and non-transformed RWPE-1 cells; PC3 and C4-2MDVR xenograft murine models; PCa patient tissue samples	1. Gln-derived nitrogen and carbon are both required for pyrimidine synthesis in PCa cells, whereas Gln is not a main carbon source for purine synthesis; 2. CAD, a key enzyme for pyrimidine synthesis, is upregulated in PCa tissues; 3. A combination of CAD knockdown and GLS inhibition with CD-839 or knockdown has a superior inhibitory effect on PCa in vitro and in vivo than inhibition of a single protein.	[21]
18	GLS (GAC and KGA isoforms), glutaminolysis, AR and c-MYC signaling	PCa cell lines LNCaP, PC3, C4-2, C4-2MDVR; PCa patient tissue samples; LNCaP and PC3 xenograft murine models	1. ADT inhibits glutaminolysis; 2. Advanced PCa and CRPC cells are highly dependent on Gln; 3. GLS isoforms are differently expressed at the different stages of PCa: KGA is highly expressed in hormone-naive PCa cells, whereas GAC is highly expressed in advanced PCa and CRPC; 4. GAC and KGA expression levels inversely correlate in PCa tissues; 5. GLS isoform switch is associated with PCa resistance to ADT; 6. Advanced PCa and CRPC cells are more sensitive to GLS inhibition with CB-839 in vitro and in vivo than androgen-dependent PCa; 7. GLS isoform switch is regulated by MYC and AR.	[121]
19	L-ASP, Gln transporters, Asn catabolism, cell cycle and DNA repair signaling	PCa cell lines 22Rv1, PC3, ARCaP_M_ and its radiation-resistant derivative ARCaP_M_-IR; CAF; ARCaP_M_/CAF xenograft murine models	1. Gln is conditionally essential for PCa cells; 2. L-ASP sensitizes PCa cells to radiotherapy through depletion of Gln; 3. Both L-ASP and Gln depletion induce cell cycle arrest and inhibit DNA repair; 4. CAF-induced PCa radioresistance can be decreased in vitro and in vivo by L-ASP.	[104]
20	Pro and Gln biosynthesis pathways, P5CS, ALDH18A1; TCA cycle, pyrimidine synthesis	PCa cell lines VCAP, 22Rv1, LNCaP and PC3; multiple cancer cell lines; gastric cancer patient tissue samples	1. α-KG, Asn, and nucleotides are the key metabolites for cell survival under Gln deprivation; 2. A lowering of P5CS (ALDH18A1) expression is a common adaptation to Gln deprivation in different types of tumor cells, including PCa; 3. P5CS inhibition promotes Gln de novo synthesis.	[143]
21	GLS, glutaminolysis, mitochondrial bioenergetics, cell cycle and viability regulation	Docetaxel-sensitive and -resistant PCa cell lines PC3 and DU145; PCa patient tissue samples	1. Gln deprivation and GLS inhibition with CB-839 reduces mitochondrial functions and induces apoptosis in chemotherapy-resistant and sensitive PCa cells; 2. Docetaxel-resistant PCa cells are more sensitive to the Gln metabolism inhibition than parental cells; 3. GLS expression is elevated in PCa and correlates with clinical outcomes.	[144]

Acronyms: ADT: androgen-deprivation therapy; ALDH18A1: aldehyde dehydrogenase 18 family member A1; ALKBH: alpha-ketoglutarate-dependent dioxygenase; AR: androgen receptor; ASCT2: alanine, serine, cysteine transporter 2; Asn: asparagine; BPH: benign prostate hyperplasia CAD: carbamoyl-phosphate synthase (CAD); CAFs: cancer-associated fibroblasts; CSCs: cancer stem cells; CRPC: castration-resistant PCa; FA: fatty acids; Gln: glutamine; GLS: glutaminase; L-ASP: L-asparaginase; MEF: mouse embryonic fibroblasts; miR: micro RNA; MMS: methyl methanesulfonate; mTORC1: mammalian target of rapamycin complex 1; OCR: oxygen consumption rate; P5CS: pyrroline-5-carboxylate synthase; PCa: prostate cancer; PDHA1: pyruvate dehydrogenase A1; POX/PRODH: proline oxidase/dehydrogenase; PPP: pentose phosphate pathway; Pro: proline; PTEN: phosphatase and tensin homolog; RASAL3: Ras activating protein-like 3; TCA cycle: tricarboxylic acid cycle; TXNIP: thioredoxin interacting protein.
